# The Triglyceride-Glucose Index Predicts Coronary Artery Disease Severity and Cardiovascular Outcomes in Patients with Non-ST-Segment Elevation Acute Coronary Syndrome

**DOI:** 10.1155/2019/6891537

**Published:** 2019-06-11

**Authors:** Qi Mao, Denglu Zhou, Youmei Li, Yuqing Wang, Shang-Cheng Xu, Xiao-Hui Zhao

**Affiliations:** ^1^Department of Cardiovascular Medicine, Institute of Cardiovascular Research, Xinqiao Hospital, Army Medical University, Chongqing 400037, China; ^2^Department of Occupational Health, Army Medical University, Chongqing 400038, China

## Abstract

Non-ST-segment elevation acute coronary syndrome (NSTE-ACS) is the leading cause of morbidity and mortality from cardiovascular disease worldwide. Several recent studies have shown the relationship between the triglyceride-glucose (TyG) index and vascular disease; however, the role of the TyG index in NSTE-ACS has not been extensively assessed. Thus, we aimed to investigate the association of the TyG index with cardiovascular risk factors and outcomes in NSTE-ACS. Overall, 438 patients with NSTE-ACS were enrolled to examine the association of the TyG index with the SYNTAX score and major adverse cardiovascular events (MACEs). The TyG index was calculated as ln (fasting triglyceride (mg/dL) × fasting glucose (mg/dL)/2). The severity of coronary lesions was quantified by the SYNTAX score. MACEs included cardiac death, nonfatal myocardial infarction, target vessel revascularization, congestive heart failure, and nonfatal stroke. All the patients underwent a 12-month follow-up for MACEs after admission. Multivariate regression analysis identified metabolic risk factors as independent parameters correlated with the TyG index. The prevalence of glucose metabolism disorder, metabolic syndrome, and MACEs increased with increasing TyG index. The TyG index showed a strong diagnostic performance for cardiovascular risk factors and was independently associated with the SYNTAX score (OR 6.055, 95% CI 2.915–12.579, *P* < 0.001). The risk of MACEs (12.8% and 22.8% for the low TyG index and high TyG index groups, respectively; adjusted HR = 1.791, 95% CI 1.045–3.068, *P* = 0.034) significantly increased in the high TyG index group as compared with the low TyG index group. The multivariate Cox regression analysis further revealed that the TyG index was an independent predictor of MACEs (HR 1.878, 95% CI 1.130–3.121, *P* = 0.015). In conclusion, the TyG index might be an independent predictor of coronary artery disease severity and cardiovascular outcomes in NSTE-ACS.

## 1. Introduction

Non-ST-segment elevation acute coronary syndrome (NSTE-ACS) is the leading cause of morbidity and mortality from cardiovascular disease worldwide [1–3]. Therefore, it is crucial to identify patients at high risk of developing future adverse cardiovascular events that may contribute to optimal management.

Insulin resistance (IR) is a hallmark of metabolic syndrome (MetS) and is considered to be a pivotal risk factor for cardiometabolic diseases [4, 5]. A high IR level not only is associated with increasing risk of developing cardiovascular disease (CVD) but also is significantly associated with high risk of cardiovascular outcomes [6, 7]. However, direct measurement methods of IR (the hyperinsulinemic euglycemic glucose clamp and the insulin suppression test) are invasive, costly, and complicated procedures [8]. Simple and accessible markers of IR are required for epidemiological study and clinical practice.

High levels of triglyceride (TG) and fasting blood glucose (FBG) are the components of MetS, which is one of the most important risk factors for CVD [4]. The combination of both indicators, the triglyceride-glucose (TyG) index, has been reported to be significantly correlated with IR and has been proposed as a reliable surrogate marker of IR [9]. However, most of the relevant studies focused on the impact of the TyG index on metabolic diseases [10–12]. Although several recent studies have showed the association of the TyG index with vascular disease, no studies have further explored the role of the TyG index in NSTE-ACS [13, 14]. Therefore, in this study, we aimed to investigate the correlation between the TyG index and cardiovascular risk factors and examine the association of the TyG index with cardiovascular outcomes in NSTE-ACS.

## 2. Materials and Methods

### 2.1. Study Population

The study complied with the Declaration of Helsinki and was approved by the Ethics Review Committee of Xinqiao Hospital, Army Medical University (Chongqing, China). All patients provided informed consent.

This was an observational study involving patients diagnosed with NSTE-ACS who were admitted between January 2017 and September 2017 in our institution. A total of 791 consecutive patients with NSTE-ACS were examined. The inclusion criteria were as follows: (1) with complete clinical information, (2) underwent coronary angiography, and (3) estimated glomerular filtration rate (eGFR) ≥ 60 mL/min∗1.73 m^2^ at admission. The exclusion criteria were as follows: nonobstructive coronary disease, primary cardiomyopathy, valvular heart disease, severe hepatic dysfunction, significant infection, thyroid and adrenal cortex dysfunction, autoimmune diseases, hematologic disorders, malignant diseases, and surgery or trauma 3 months prior to participation. In addition, patients taking statins and triglyceride-lowering medication before the onset of NSTE-ACS were excluded. Finally, a cohort of 438 patients with NSTE-ACS was enrolled.

### 2.2. Data Collection and Follow-Up

Clinical data were collected from medical records by trained clinicians. These included demographic data, medical history, laboratory indicators, and basic medication information. The venous blood samples were collected after overnight fasting before coronary angiography. Routine biochemical parameters including lipids, blood glucose, and renal function were assayed using a Beckman Coulter DXC800 system (USA). The angiographic data were obtained from the cardiac catheterization laboratory records. The SYNTAX score for quantifying the severity of coronary lesions was calculated by experienced interventional cardiologists using the score calculator (version 2.28) in the SYNTAX score website. Major adverse cardiovascular events (MACEs) were defined as the composite of cardiac death, nonfatal myocardial infarction, target vessel revascularization (TVR), congestive heart failure, and nonfatal stroke. All patients had a 12-month follow-up for MACEs after admission, and follow-up data were obtained from hospital records or by interviewing (in person or by telephone) patients and their families.

### 2.3. Definition

The definition of NSTE-ACS complied with the current guideline of the European Society of Cardiology (ESC) [15]. Calculation of the Global Registry of Acute Coronary Events (GRACE) risk score was based on clinical history, electrocardiogram, and laboratory parameters at admission [16]. Multivessel disease was defined as at least 2-vessel disease or left main disease with >50% luminal narrowing [17]. The SYNTAX score was determined by all coronary lesions with >50% diameter stenosis in a vessel > 1.5 mm [18]. The basic drug treatment for the NSTE-ACS patients was in compliance with the current guidelines [15]. Percutaneous coronary intervention (PCI) was determined by experienced cardiologists based on individual risk and decisions from patients. Glucose metabolism disorder (GMD) was defined as a fasting glucose concentration ≥ 5.6 mmol/L (100 mg/dL) and/or diabetes mellitus [19]. Metabolic syndrome (MetS) was defined according to the National Cholesterol Education Program's Adult Treatment Panel III (NCEP ATP III) criteria [20].

The TyG index was calculated as ln (fasting triglycerides (mg/dL) × fasting glucose (mg/dL)/2) [9]. Serum creatinine was used to obtain the estimated glomerular filtration rate (eGFR) according to the Modification of Diet in Renal Disease (MDRD) Study equation: eGFR (mL/min∗1.73 m^2^) = 186 × Scr^−1.154^ × age^−0.203^ × 1.233 × 0.742 (if female) [21]. C-reactive protein (CRP) and B type natriuretic peptide (BNP) were, respectively, converted into binary categorical variables by 5 *μ*g/mL as the cutoff value of the elevated CRP level and 100 pg/mL as the cutoff value of the elevated BNP level [22, 23]. SYNTAX ≥ 23 was considered as a high SYNTAX score, and GRACE ≥ 89 was also deemed as a high GRACE score [16, 18].

### 2.4. Statistical Analysis

Continuous variables were presented as the mean ± SD or median (IQR) according to the presence or absence of normal distribution, and categorical data were expressed as frequencies and percentages. To compare the baseline characteristics, the patients were divided into the following groups by the median TyG index level (8.805) of the cohort: low TyG index group (≤8.805) and high TyG index group (>8.805). Differences in continuous variables classified by the median of the TyG index were assessed by the Mann-Whitney *U* test or *t*-test as appropriate, and differences in continuous variables stratified by the TyG index level and GMD were assessed by the Kruskal-Wallis *H* test. Differences in categorical variables were evaluated by the Chi-square test or the Fisher exact test. Correlation between the TyG index and other parameters was assessed using the Spearman rank correlation test. Diagnostic performances of the TyG index on clinical variables were assessed by receiver operating characteristic (ROC) curve analysis. Linear regression analyses were performed to reveal the factors associated with the TyG index, and the selection of variables was made based on a forward stepwise method in the multivariate model. Logistic regression analysis was applied to evaluate the independent predictors of a high SYNTAX score (≥ 23), and the variables with unadjusted *P* value of <0.1 were selected as potential risk factors and included in the multivariate model. Event-free survival time was the period from the date of admission to the date of cardiovascular events as verified during the follow-up. Survival curves or cumulative hazard curves for cardiovascular outcomes were constructed using the Kaplan-Meier method with adjustment in a Cox proportional hazards model. Univariate and multivariate Cox regression analyses were performed to assess the association between the TyG index and cardiovascular outcomes, and risk factors that were chosen for their clinical importance as well as statistical significance (*P* < 0.05) were used as variables in a multivariate Cox model. *P* < 0.05 was considered statistically significant. All statistical analyses were performed using SPSS software version 22.0 (SPSS Inc., Chicago, Illinois).

## 3. Results

### 3.1. Baseline Characteristics

The characteristics of the study population are described in Table 1. Patients in the high TyG index group were older than those in the low TyG index group (*P* < 0.001). The patients with a high TyG index had a higher incidence of diabetes (*P* < 0.001), GMD (*P* < 0.001), and MetS (*P* < 0.001), had more severe coronary artery lesion (*P* < 0.001), and had a higher GRACE score (*P* < 0.001). Significant differences in laboratory parameters were also observed in the two groups. Importantly, more MACEs were found in the high TyG index group (*P* = 0.006).

### 3.2. Correlation between the TyG Index and Cardiovascular Risk Factors

Spearman rank correlation analysis was used to examine the relationship between the TyG index and important variables including cardiovascular risk factors. The TyG index was significantly correlated with age, body mass index (BMI), LDL-C, HDL-C, TG/HDL-C ratio, LDL-C/HDL-C ratio, HbA1c, uric acid, BNP, CRP, SYNTAX score, and GRACE score (Table 2).

### 3.3. Evaluation of Factors Associated with the TyG Index

Univariate linear regression showed that the TyG index level associated positively with BMI, GMD, LDL-C, uric acid, BNP, and CRP and negatively correlated with HDL-C and eGFR-MDRD. Multivariate analysis further indicated that BMI (*β* = 0.018, *P* = 0.012), GMD (*β* = 0.417, *P* < 0.001), LDL-C (*β* = 0.172, *P* < 0.001), HDL-C (*β* = −0.335, *P* < 0.001), uric acid (*β* = 0.001, *P* = 0.002), and CRP (*β* = 0.017, *P* < 0.001) were independently associated with the TyG index (Table 3).

### 3.4. Comparisons of Cardiovascular Risk Factors and Outcomes according to Restratification Based on the TyG Index Level and GMD

**Figure 1:**
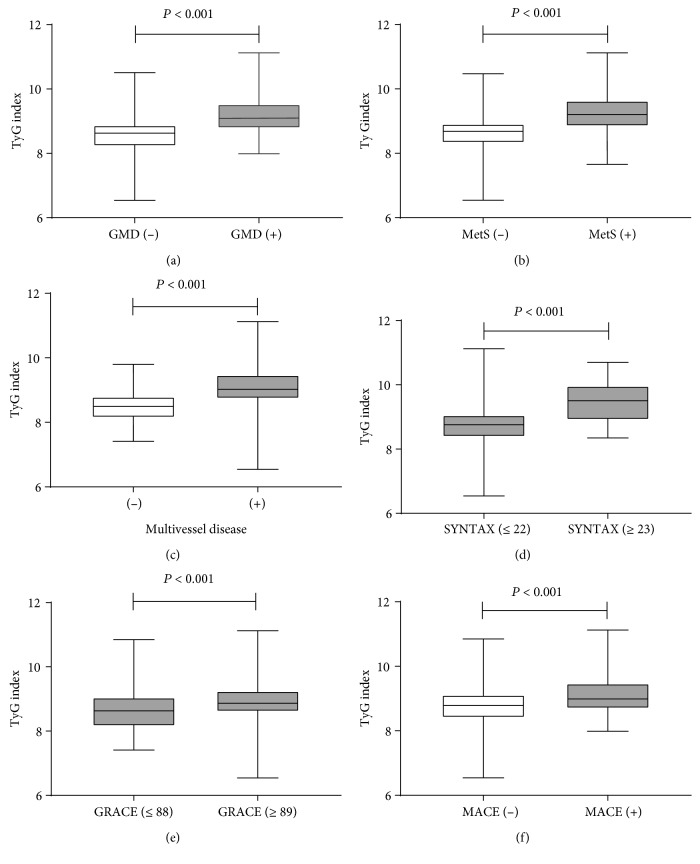
Comparisons of the TyG index levels in the NSTE-ACS patients with or without GMD (a), metabolic syndrome (b), multivessel disease (c), high SYNTAX score (d), high GRACE score (e), and MACE (f). Abbreviations: GMD: glucose metabolism disorder; MetS: metabolic syndrome; MACEs: major adverse cardiovascular events.

Furthermore, we stratified the NSTE-ACS patients with or without GMD into the following four groups based on the TyG index levels: low TyG index/GMD (-) (*n* = 168), low TyG index/GMD (+) (*n* = 51), high TyG index/GMD (-) (*n* = 80), and high TyG index/GMD (+) (*n* = 139). An elevated CRP level, elevated BNP level, high GRACE score, multivessel disease, high SYNTAX score, MetS, and MACEs were significantly different among the four groups (*P* < 0.001). The high TyG index/GMD (+) group had more cardiovascular risk factors and events than the other three groups (Table 4). In addition, comparisons of the TyG index levels in the NSTE-ACS population with or without GMD, multivessel disease, high SYNTAX score, high GRACE score, MetS, and MACEs are shown in Figure 1.

### 3.5. Diagnostic Performance of the TyG Index for Cardiovascular Risk Factors and Outcomes

In the ROC curve analysis, the cutoff values of the TyG index were constructed according to the ROC curves for identifying the patients with more cardiometabolic risk factors and for predicting the occurrence of MACEs and its components. According to the area under the curve (AUC), the TyG index was shown to be a powerful diagnostic indicator of cardiovascular risk factors and outcomes. Using the cutoff points, the predictive cutoff value of the TyG index for MACEs was 8.556 (AUC 0.639, 95% CI 0.574–0.703, *P* < 0.001) (Table 5).

### 3.6. Association of the TyG Index with High SYNTAX Scores

Univariate logistic regression analysis showed that age, MetS, LDL-C, HDL-C, eGFR-MDRD, Killip class > 1, GRACE score ≥ 89, CRP, and TyG index were potential risk factors for a high SYNTAX score. These potential risk factors from the univariate analysis were used as variables in the multivariate model, and the results revealed that the TyG index was an independent predictor of the high SYNTAX score (OR 6.055, 95% CI 2.915–12.579, *P* < 0.001) (Table 6).

### 3.7. Association of the TyG Index with Outcomes

The Cox proportional hazards model was applied to examine the association between the TyG index and MACEs. Univariate analysis showed that the TyG index was significantly associated with MACEs (HR 1.951, 95% CI 1.416–2.688, *P* < 0.001). Several risk factors including important clinical and significant variables in the univariate model were included in the multivariate model for adjustment, and the TyG index also remained to be an independent predictor of MACEs in adjusted models 1 (HR 1.970, 95% CI 1.431–2.712, *P* < 0.001) and 2 (HR 1.878, 95% CI 1.130–3.121, *P* = 0.015) (Table 7).

### 3.8. Kaplan-Meier Survival Analysis for Cardiovascular Outcomes

**Figure 2:**
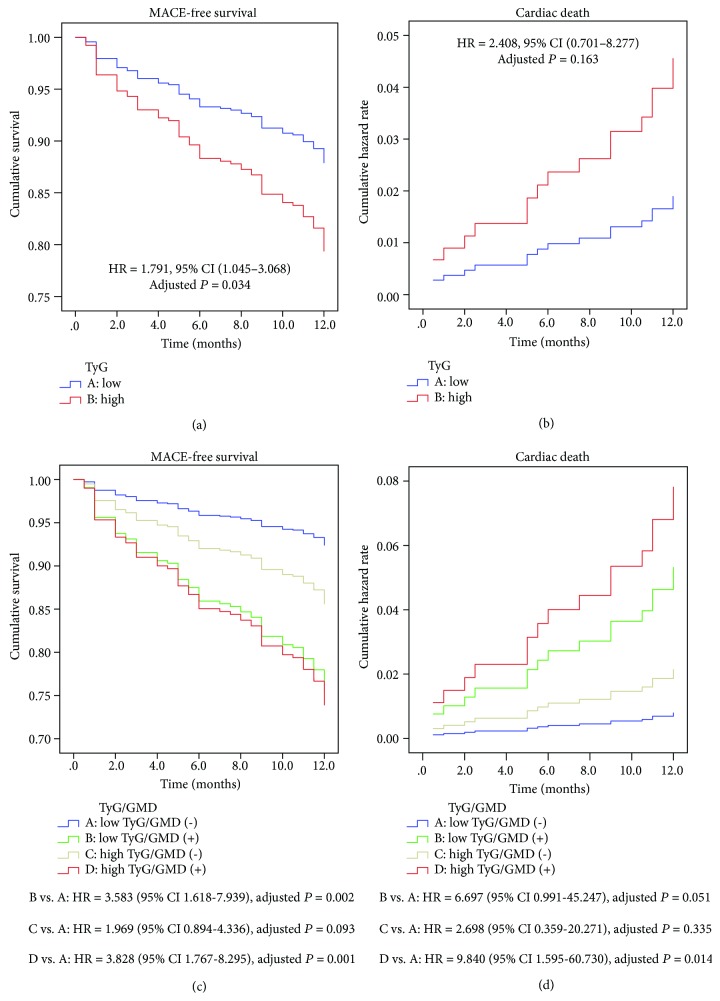
Kaplan-Meier curves for survival analysis of MACE-free survival (a, c) according to the level of the TyG index with or without GMD. Cumulative hazard curves of cardiac death (b, d) according to the level of the TyG index with or without GMD. Adjusted for age, gender, metabolic syndrome, LDL-C, HDL-C, SYNTAX score, CRP, basal insulin, sulfonylurea, metformin, *α*-glucosidase inhibitor, ACEI/ARB, beta-blocker, and PCI/CABG. Abbreviations: GMD: glucose metabolism disorder; MACEs: major adverse cardiovascular events.

During the 12-month follow-up, MACEs occurred in 28 (12.8%) patients in the low TyG index group and 50 (22.8%) patients in the high TyG index group (adjusted HR = 1.791, 95% CI 1.045–3.068, *P* = 0.034). Cardiac death occurred in 5 (2.3%) patients in the low TyG index group and 13 (5.9%) patients in the high TyG index group (adjusted HR = 2.408, 95% CI 0.701–8.277, *P* = 0.163). After GMD stratification, there were significant between-group differences in MACEs (15 patients (8.9%) in the low TyG index group without GMD vs. 37 patients (26.6%) in the high TyG index group with GMD; *P* < 0.001) and in cardiac death (2 patients (1.2%) in the low TyG index group without GMD vs. 11 patients (7.9%) in the high TyG index group with GMD; *P* = 0.008). In a post hoc analysis, there were more MACEs (adjusted HR = 3.828, 95% CI 1.767–8.295, *P* = 0.001) and cardiac death (adjusted HR = 9.840, 95% CI 1.595–60.730, *P* = 0.014) in the high TyG index group with GMD than the low TyG index group without GMD (Figure 2).

## 4. Discussion

To the best of our knowledge, this is the first study to examine the association of the TyG index with outcomes in a NSTE-ACS population. The main findings are as follows: (1) the TyG index is correlated with multiple cardiovascular risk factors and (2) the TyG index is an independent predictor of coronary artery disease severity and MACEs.

The TyG index is a composite indicator composed of TG and FBG and is demonstrated to be a good marker of IR and a predictor of type 2 diabetes mellitus (T2DM) [10, 12]. As of yet, only a few studies on the relationship between the TyG index and CVD have been reported. Sánchez-Íñigo et al. [24] noted that the TyG index was significantly associated with high risk of developing CVD and a good predictor for the Framingham model in the VMCUN cohort. However, Vega et al. [25] showed that the TyG index was only a predictor of T2DM rather than CVD compared to the TG/HDL-C ratio, which was deemed as a marker of metabolism disorder. This might be attributed to the younger age, dietary difference, ethnicity variation, low incidence of MetS, and less other cardiovascular risk factors in that study population. Importantly, the prevalence of abnormal glucose metabolism such as fasting blood glucose and diabetes was obviously reduced while non-HDL-C significantly increased in the study population of Vega et al. compared to the study by Sánchez-Íñigo et al. The predictive effect of the TyG index on CVD risk might depend on the reflection to glucose and lipid metabolism disorders. The TyG index was still a predictor of CVD without non-HDL-C adjustment in the study of Vega et al. Of note, both of these two studies were performed mainly in the Caucasian population and for the purpose of primary prevention. Our results showed that the high TyG index group had a significant higher incidence of MACEs at 12 months of follow-up, indicating an independent prognostic role of the TyG index for the NSTE-ACS population.

Although the mechanism underlying the relationship between the TyG index and NSTE-ACS is unknown, the TyG index has been deemed as a useful atherogenic indicator linked to IR and MetS [26]. A series of studies have demonstrated a strong association between the TyG index and IR, diabetes, hypertension, MetS, and atherosclerosis [24, 27, 28]. Consistent with previous studies, our results also revealed the correlation of the TyG index with metabolic risk factors in NSTE-ACS, most of which were the components of MetS and risk factors of atherosclerosis. Moreover, the TyG index presented a high correlation with the TG/HDL-C ratio which was also a marker of IR. These findings reconfirmed the correlation of the TyG index with metabolism disorder and IR. Furthermore, the TyG index showed a strong diagnostic performance for GMD and MetS in the ROC analysis. This might be helpful for the prediction of diabetes and contribute to early identification of individuals at high risk of developing MACEs.

The relationship between the TyG index and coronary artery disease severity remains unclear in NSTE-ACS. In recent studies, Lee et al. [13] and Kim et al. [14], respectively, reported that the TyG index was independently associated with arterial stiffness and coronary artery calcification in Korean adults; Zheng and Mao [29] found that the TyG index was a predictor of hypertension in a Chinese population. These studies showed that the TyG index might serve as a biomarker for vascular disease and implied that IR reflected by the TyG index might have participated in the process of vascular remodeling and atherogenesis. Although Lee et al. [30] reported that the TyG index was associated with risk of coronary artery stenosis in asymptomatic patients with T2DM, this study was mainly based on a diabetes population, and the severity of coronary artery stenosis was not quantified. In addition, Lambrinoudaki et al. [31] found that the TyG index was associated with carotid atherosclerosis, but this study mainly focused on subclinical vascular disease in postmenopausal women. In our study, the number of diseased vessels and the SYNTAX score increased with increasing TyG index level, and the association between the TyG index and the SYNTAX score was related to cardiometabolic risk factors, such as older age, BMI, inflammation, GMD, and MetS. This suggested that higher IR represented by the TyG index makes the patients more susceptible to CVD risk factors thus causing atherosclerosis. Further analysis showed that the TyG index might serve as a marker of severity of coronary artery stenosis and was independently associated with the SYNTAX score. The proatherosclerosis mechanisms of the TyG index may be ascribed to systemic inflammation, endothelial dysfunction, oxidative stress, and vascular remodeling mediated by IR [5, 26, 32].

Previous evidence showed that patients with metabolism disorder had higher MACE rates and more severe coronary artery lesion in the ACS population [33, 34]. In the current study, the patients with GMD consistently had more cardiovascular events regardless of the TyG index level, but the patients with a high TyG index still had a higher incidence of MACEs in the non-GMD population. The results suggested that the TyG index might be independent of GMD to influence cardiovascular outcomes. Multivariate analysis further indicated that the TyG index was an independent predictor of MACEs after adjustment for multiple risk factors. The predictive effect of the TyG index on cardiovascular outcomes should be interpreted as IR reflected by the TyG index. IR may be the mechanism in developing MACEs in the NSTE-ACS population. First, IR may increase sympathetic activity, secretion of catecholamines, and myocardial oxygen consumption [35, 36]. Second, IR may activate the renin-angiotensin-aldosterone system, accelerate ventricular remodeling, promote water and sodium reabsorption, increase circulation capacity, and ultimately cause cardiac insufficiency [37, 38]. Third, IR may increase the production of clotting and inflammatory factors, contribute to coagulation imbalance and disturbances of fibrinolysis, and eventually contribute to thrombosis [39]. Fourth, IR may change cardiac metabolism, contribute to energy production defect, impair contractile function, and thus lead to cardiomyopathy and heart failure [40]. Fifth, IR may promote atherosclerosis progression, induce plaque instability, and therefore contribute to increase MACEs [41–43].

Our study had several limitations. First, this was a single center, observational study with potential selection bias. Second, the sample size was relatively small, and the clinical follow-up duration was short, which might have an influence on the reliability of results. Third, laboratory parameters were only measured once at admission with a potential bias due to measurement error. Fourth, we did not calculate the insulin resistance index of homeostasis model assessment (HOMA-IR); thus, we could not compare the role of the TyG index and HOMA-IR in NSTE-ACS. Finally, residual compounding factors and unrecorded variables such as life style and nutritional data might also affect outcomes.

## 5. Conclusion

Our study showed that the TyG index independently predicted coronary artery disease severity and cardiovascular outcomes in NSTE-ACS. The detection of the TyG index might be beneficial for early stratification and intervention to prevent MACEs. Further large-scale studies will be required to evaluate the implication of these results.

## Figures and Tables

**Table 1:** Baseline characteristics of the study population.

Variables	Total	TyG index		*P* value
	*N* = 438	Low (≤8.805, *n* = 219)	High (>8.805, *n* = 219)	
Age (years)	62.5 (53.0–68.0)	60.0 (53.0–67.0)	64.0 (54.0–71.0)	0.001
Male, *n* (%)	295 (67.4)	156 (71.2)	139 (63.5)	0.083
BMI ≥ 24 kg/m^2^, *n* (%)	231 (52.7)	105 (47.9)	126 (57.5)	0.044
BMI (kg/m^2^)	24.33 ± 3.17	23.90 ± 2.96	24.76 ± 3.32	0.005
Smoking, *n* (%)	194 (44.3)	101 (46.1)	93 (42.5)	0.442
Hypertension, *n* (%)	137 (31.3)	66 (30.1)	71 (32.4)	0.606
Diabetes, *n* (%)	143 (32.6)	29 (13.2)	114 (52.1)	<0.001
Glucose metabolism disorder, *n* (%)	190 (43.4)	51 (23.3)	139 (63.5)	<0.001
Stroke history, *n* (%)	47 (10.7)	21 (9.6)	26 (11.9)	0.440
Metabolic syndrome, *n* (%)	139 (31.7)	33 (15.1)	106 (48.4)	<0.001
Killip class > 1, *n* (%)	129 (29.5)	52 (23.7)	77 (35.2)	0.009
Multivessel disease, *n* (%)	260 (59.4)	82 (37.4)	178 (81.3)	<0.001
SYNTAX score	9.5 (5.0–18.0)	6.0 (3.0–9.5)	15.0 (9.5–22.0)	<0.001
GRACE score for 6 months	100.0 (81.0–122.0)	92.0 (76.0–112.0)	107.0 (88.0–130.0)	<0.001
*Laboratory parameters*				
TyG index	8.805 (8.491–9.191)	8.492 (8.147–8.661)	9.189 (8.957–9.549)	<0.001
HbA1c (%)	6.1 (5.7–6.6)	5.8 (5.5–6.2)	6.4 (5.9–7.2)	<0.001
FBG (mmol/L)	5.29 (4.67–6.19)	4.82 (4.38–5.32)	5.89 (5.16–7.28)	<0.001
TG (mmol/L)	1.60 (1.16–2.01)	1.16 (0.91–1.50)	1.98 (1.72–2.78)	<0.001
LDL-C (mmol/L)	2.66 (2.12–3.18)	2.49 (1.99–2.99)	2.84 (2.34–3.40)	<0.001
HDL-C (mmol/L)	0.99 (0.84–1.16)	1.01 (0.85–1.22)	0.98 (0.84–1.12)	0.045
TG/HDL-C ratio	1.580 (1.120–2.193)	1.170 (0.824–1.506)	2.103 (1.664–2.878)	<0.001
LDL-C/HDL-C ratio	2.683 (2.095–3.251)	2.444 (1.875–3.011)	2.949 (2.438–3.520)	<0.001
Uric acid (*μ*mol/L)	348.3 ± 94.8	334.9 ± 86.6	361.7 ± 100.7	0.003
Creatinine (*μ*mol/L)	72.35 (62.30–81.73)	70.9 (61.1–80.5)	73.4 (63.3–82.7)	0.123
eGFR-MDRD (mL/min∗1.73 m^2^)	95.9 (80.1–108.0)	97.1 (84.3–111.6)	92.1 (77.5–104.7)	0.003
CRP (*μ*g/mL)	5.50 (5.00–9.83)	5.0 (5.0–5.2)	8.4 (5.7–16.7)	<0.001
BNP (pg/mL)	61.0 (17.4–175.3)	49.0 (10.6–145.0)	75.3 (24.5–233.0)	0.002
*Medication*				
Basal insulin, *n* (%)	21 (4.8)	3 (1.4)	18 (8.2)	0.001
Sulfonylurea, *n* (%)	32 (7.3)	7 (3.2)	25 (11.4)	0.001
Metformin, *n* (%)	91 (20.8)	16 (7.3)	75 (34.2)	<0.001
*α*-Glucosidase inhibitor, *n* (%)	27 (6.2)	7 (3.2)	20 (9.1)	0.010
ACEI/ARB, *n* (%)	363 (82.9)	180 (82.2)	183 (83.6)	0.704
Beta-blocker, *n* (%)	322 (73.5)	159 (72.6)	163 (74.4)	0.665
PCI/CABG, *n* (%)	334 (76.3)	169 (77.2)	165 (75.3)	0.653
*Outcomes*				
In-hospital cardiovascular mortality, *n* (%)	3 (0.7)	1 (0.5)	2 (0.9)	1
MACEs, *n* (%)	78 (17.8)	28 (12.8)	50 (22.8)	0.006
Cardiac death, *n* (%)	18 (4.1)	5 (2.3)	13 (5.9)	0.054
Nonfatal myocardial infarction, *n* (%)	20 (4.6)	8 (3.7)	12 (5.5)	0.360
TVR, *n* (%)	24 (5.5)	8 (3.7)	16 (7.3)	0.093
Congestive heart failure, *n* (%)	10 (2.3)	5 (2.3)	5 (2.3)	1
Nonfatal stroke, *n* (%)	6 (1.4)	2 (0.9)	4 (1.8)	0.681

Data are expressed as the mean ± SD, median (IQR), or *n* (%). Abbreviations: BMI: body mass index; TyG index: triglyceride-glucose index; FBG: fasting blood glucose; TG: triglyceride; LDL-C: low-density lipoprotein cholesterol; HDL-C: high-density lipoprotein cholesterol; eGFR-MDRD: estimated glomerular filtration rate based on the MDRD equation; PCI: percutaneous coronary intervention; CABG: coronary artery bypass grafting; ACEI: angiotensin-converting enzyme inhibitors; ARB: angiotensin receptor blocker; CRP: C-reactive protein; BNP: B type natriuretic peptide; MACEs: major adverse cardiovascular events; TVR: target vessel revascularization.

**Table 2:** Correlation between the TyG index and clinical variables.

	*R* (Spearman)	*P* value
Age	0.141	0.003
BMI	0.204	<0.001
TG/HDL-C ratio	0.806	<0.001
LDL-C/HDL-C ratio	0.388	<0.001
LDL-C	0.303	<0.001
HDL-C	-0.135	0.005
HbA1c	0.456	<0.001
Uric acid	0.175	<0.001
CRP	0.646	<0.001
BNP	0.158	0.001
SYNTAX score	0.658	<0.001
GRACE score	0.301	<0.001

Abbreviations: BMI: body mass index; TG: triglyceride; LDL-C: low-density lipoprotein cholesterol; HDL-C: high-density lipoprotein cholesterol; CRP: C-reactive protein; BNP: B type natriuretic peptide.

**Table 3:** Univariate and multivariate linear regression analyses for the TyG index.

Variable	Univariate			Multivariate		
	*β*	Standard *β*	*P* value	*β*	Standard *β*	*P* value
Age	0.003	0.044	0.362			
Male	-0.055	-0.041	0.388			
BMI	0.041	0.209	<0.001	0.018	0.093	0.012
Smoking	0.035	0.028	0.565			
Hypertension	0.060	0.045	0.351			
GMD	0.590	0.466	<0.001	0.417	0.330	<0.001
LDL-C	0.220	0.281	<0.001	0.172	0.220	<0.001
HDL-C	-0.398	-0.178	<0.001	-0.335	-0.150	<0.001
Uric acid	0.001	0.197	<0.001	0.001	0.114	0.002
eGFR-MDRD	-0.004	-0.133	0.005	—	—	—
BNP	0.001	0.139	0.004	—	—	—
CRP	0.026	0.480	<0.001	0.017	0.321	<0.001

Abbreviations: BMI: body mass index; GMD: glucose metabolism disorder; LDL-C: low-density lipoprotein cholesterol; HDL-C: high-density lipoprotein cholesterol; eGFR-MDRD: estimated glomerular filtration rate based on the MDRD equation; CRP: C-reactive protein; BNP: B type natriuretic peptide.

**Table 4:** Cardiovascular risk factors and outcomes.

	Low TyG index (≤8.805, *n* = 219)		High TyG index (>8.805, *n* = 219)		*P* value
	GMD (-), *n* = 168	GMD (+), *n* = 51	GMD (-), *n* = 80	GMD (+), *n* = 139	
CRP > 5 *μ*g/mL, *n* (%)	43 (25.6)	17 (33.3)	57 (71.3)	122 (87.8)	<0.001
BNP > 100 pg/mL, *n* (%)	47 (28.0)	24 (47.1)	24 (30.0)	70 (50.4)	<0.001
Multivessel disease, *n* (%)	55 (32.7)	27 (52.9)	59 (73.8)	119 (85.6)	<0.001
SYNTAX score	5.0 (3.0–8.0)	7.0 (5.0–13.0)	12.0 (8.0–17.0)	17.0 (12.0–25.0)	<0.001
GRACE score	87.0 (74.0–105.0)	105.0 (88.0–122.0)	105.0 (82.0–120.0)	113.0 (92.0–135.0)	<0.001
MetS, *n* (%)	18 (10.7)	15 (29.4)	27 (33.8)	79 (56.8)	<0.001
MACEs, *n* (%)	15 (8.9)	13 (25.5)	13 (16.3)	37 (26.6)	<0.001
Cardiac death, *n* (%)	2 (1.2)	3 (5.9)	2 (2.5)	11 (7.9)	0.014
Nonfatal myocardial infarction, *n* (%)	3 (1.8)	5 (9.8)	4 (5.0)	8 (5.8)	0.056
TVR, *n* (%)	5 (3.0)	3 (5.9)	4 (5.0)	12 (8.6)	0.178
Congestive heart failure, *n* (%)	4 (2.4)	1 (2.0)	2 (2.5)	3 (2.2)	1
Nonfatal stroke, *n* (%)	1 (0.6)	1 (2.0)	1 (1.3)	3 (2.2)	0.488

Data are expressed as the median (IQR) or *n* (%). Abbreviations: MACEs: major adverse cardiovascular events; TVR: target vessel revascularization; GMD: glucose metabolism disorder; MetS: metabolic syndrome; CRP: C-reactive protein; BNP: B type natriuretic peptide.

**Table 5:** Summary of ROC curves.

	AUC	95% CI	*P* value	TyG index cutoff	Sensitivity	Specificity	Youden index
CRP > 5 *μ*g/mL	0.848	0.812–0.884	<0.001	8.813	0.745	0.809	0.554
BNP > 100 pg/mL	0.575	0.520–0.630	0.008	9.164	0.352	0.795	0.146
GMD	0.778	0.735–0.822	<0.001	8.946	0.647	0.823	0.470
MetS	0.779	0.732–0.827	<0.001	8.925	0.712	0.756	0.468
Multivessel disease	0.802	0.762–0.843	<0.001	8.819	0.673	0.787	0.460
SYNTAX score ≥ 23	0.811	0.752–0.870	<0.001	9.299	0.687	0.876	0.563
GRACE score ≥ 89	0.631	0.574–0.687	<0.001	8.530	0.796	0.462	0.258
MACEs	0.639	0.574–0.703	<0.001	8.556	0.872	0.353	0.225
Cardiac death	0.664	0.542–0.785	0.018	9.022	0.611	0.671	0.283
TVR	0.664	0.567–0.761	0.007	9.140	0.542	0.749	0.290
Nonfatal stroke	0.751	0.570–0.932	0.035	9.248	0.667	0.782	0.449

Abbreviations: MACEs: major adverse cardiovascular events; TVR: target vessel revascularization; GMD: glucose metabolism disorder; MetS: metabolic syndrome; CRP: C-reactive protein; BNP: B type natriuretic peptide.

**Table 6:** Association between the TyG index and high SYNTAX score (≥23).

	Univariate			Multivariate		
	OR	95% CI	*P* value	OR	95% CI	*P* value
Age	1.037	1.007–1.067	0.015	1.014	0.971–1.059	0.529
Male	1.276	0.743–2.193	0.377			
Smoking	1.515	0.883–2.599	0.131			
Hypertension	1.178	0.663–2.093	0.576			
MetS	2.610	1.537–4.432	<0.001	1.698	0.796–3.620	0.171
TyG index	8.127	4.783–13.808	<0.001	6.055	2.915–12.579	<0.001
LDL-C	1.498	1.093–2.053	0.012	1.062	0.680–1.658	0.792
HDL-C	0.315	0.101–0.977	0.045	0.526	0.112–2.458	0.414
Uric acid	1.001	0.998–1.004	0.424			
eGFR-MDRD	0.983	0.969–0.996	0.011	0.995	0.978–1.012	0.580
Killip class > 1	2.068	1.211–3.531	0.008	1.465	0.741–2.898	0.273
GRACE score ≥ 89	5.883	2.618–13.221	<0.001	7.653	2.119–27.641	0.002
CRP	1.093	1.065–1.122	<0.001	1.071	1.036–1.106	<0.001

Abbreviations: MetS: metabolic syndrome; LDL-C: low-density lipoprotein cholesterol; HDL-C: high-density lipoprotein cholesterol; eGFR-MDRD: estimated glomerular filtration rate based on the MDRD equation; CRP: C-reactive protein.

**Table 7:** Association between the TyG index and outcomes.

Outcome variables	Unadjusted		Adjusted model 1		Adjusted model 2	
	HR (95% CI)	*P* value	HR (95% CI)	*P* value	HR (95% CI)	*P* value
MACEs	1.951 (1.416–2.688)	<0.001	1.970 (1.431–2.712)	<0.001	1.878 (1.130–3.121)	0.015
Cardiac death	2.285 (1.195–4.371)	0.012	2.304 (1.217–4.362)	0.010	2.461 (0.852–7.110)	0.096
Nonfatal MI	1.399 (0.717–2.731)	0.325	1.409 (0.715–2.777)	0.322	2.557 (0.790–8.280)	0.117
TVR	2.258 (1.285–3.968)	0.005	2.204 (1.267–3.835)	0.005	1.904 (0.760–4.771)	0.169
Congestive heart failure	1.015 (0.374–2.753)	0.977	0.989 (0.351–2.790)	0.984	0.413 (0.083–2.056)	0.280
Nonfatal stroke	4.421 (1.559–12.536)	0.005	4.803 (1.631–14.145)	0.004	3.082 (0.714–13.309)	0.132

Univariate and multivariate Cox proportional hazards regression analyses are applied. Model 1 is adjusted for age and gender. Model 2 is adjusted for model 1, metabolic syndrome, LDL-C, HDL-C, SYNTAX score, CRP, basal insulin, sulfonylurea, metformin, *α*-glucosidase inhibitor, ACEI/ARB, beta-blocker, and PCI/CABG. Abbreviations: MACEs: major adverse cardiovascular events; MI: myocardial infarction; TVR: target vessel revascularization.

## Data Availability

The data used to support the findings of this study are available from the corresponding author upon request.
